# *RUNX1* upregulation via disruption of long-range transcriptional control by a novel t(5;21)(q13;q22) translocation in acute myeloid leukemia

**DOI:** 10.1186/s12943-018-0881-2

**Published:** 2018-08-29

**Authors:** Chi-Keung Cheng, Terry H. Y. Wong, Thomas S. K. Wan, Angela Z. Wang, Natalie P. H. Chan, Nelson C. N. Chan, Chi-Kong Li, Margaret H. L. Ng

**Affiliations:** 1 Blood Cancer Cytogenetics and Genomics Laboratory, Department of Anatomical and Cellular Pathology, Prince of Wales Hospital, The Chinese University of Hong Kong, Shatin, Hong Kong; 2 Department of Pediatrics, Prince of Wales Hospital, The Chinese University of Hong Kong, Shatin, Hong Kong; 3 State Key Laboratory in Oncology in South China, The Chinese University of Hong Kong, Shatin, Hong Kong

**Keywords:** MDS, AML, *RUNX1*, Chromosomal translocation, *cis*-regulatory element

## Abstract

*RUNX1* encodes a Runt-related transcription factor that is critical for hematopoiesis. In this study, through a combinatorial molecular approach, we characterized a novel t(5;21)(q13;q22) translocation involving *RUNX1* that was acquired during the progression of myelodysplastic syndrome to acute myeloid leukemia (AML) in a pediatric patient. We found that this translocation did not generate *RUNX1* fusion but aberrantly upregulated *RUNX1*. This upregulation was attributed to the disruption of long-range chromatin interactions between the *RUNX1* P2 promoter and a silencer in the first intron of the gene. Characterization of the silencer revealed a role of SNAG repressors and their corepressor LSD1/KDM1A in mediating the effect. Our findings suggest that chromosomal rearrangements may activate *RUNX1* by perturbing its transcriptional control to contribute to AML pathogenesis, in keeping with an emerging oncogenic role of RUNX1 in leukemia.

**Electronic supplementary material:**

The online version of this article (10.1186/s12943-018-0881-2) contains supplementary material, which is available to authorized users.

## Main text

RUNX1 is a master regulator of hematopoiesis and its activity is tightly controlled at the transcriptional and post-transcriptional levels. The *RUNX1* gene contains two functionally distinct promoters, the distal P1 and proximal P2, which are separated by a large first intron of ~ 160 kilobases (kb). *RUNX1* is commonly disrupted by chromosomal translocations in hematological malignancies but the molecular consequences have only been characterized in less than half of the cases. It is generally believed that *RUNX1* translocations generate oncogenic fusion proteins or truncate RUNX1 that interfere with wild-type RUNX1. Here, we reveal a novel mechanistic impact of a *RUNX1* translocation in an acute myeloid leukemia (AML) case transformed from myelodysplastic syndrome (MDS).

**Fig. 1:**
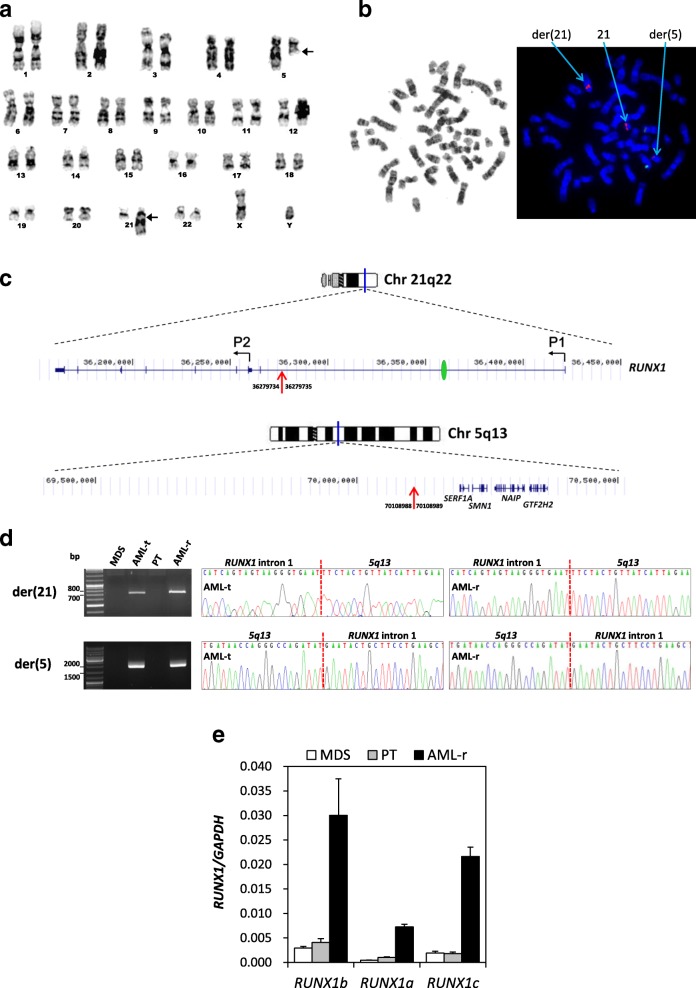
Characterization of the t(5;21) translocation breakpoints and its impact on *RUNX1* expression. **a** A representative karyotype from the relapsed AML BM of the patient revealed 46,XY,t(5;21)(q13;q22). The breakpoint regions on the derivative chromosomes 5 and 21 are arrowed. **b** FISH performed on a G-banded metaphase from the relapsed AML BM with the *ETV6*/*RUNX1* dual-color translocation probe showing *RUNX1* translocation to chromosome 5. The two green signals represent *ETV6*. **c** Chromosome 5q13 and 21q22 breakpoints mapped by WGS. The breakpoint locations (red arrows) are shown (hg19). P1 and P2 represent the two *RUNX1* promoters. The green oval represents the silencer identified in this study. **d** Breakpoint-specific PCR and Sanger sequencing revealed identical breakpoint sequences in the MDS-transformed (AML-t) and relapsed (AML-r) AML BM samples. No PCR product was obtained from the initial MDS and post-transplant (PT) BM. **e** *RUNX1* mRNA levels in BM collected at different disease states (MDS, PT and AML-r). RNA extracted from the MDS-transformed AML BM was of unsuitable quality for expression studies. Results are expressed as mean ± standard error (SE) from three independent experiments

The patient was a 2-year-old boy who presented with pallor, bruising and petechiae for one month. No hepatosplenomegaly was noted. Blood tests showed anemia, thrombocytopenia and a leucoerythroblastic picture. Bone marrow (BM) examination showed focally prominent blastic infiltrations (15–20%) with dysplastic changes involving the erythroid and megakaryocytic lineages, suggestive of the diagnosis of advanced MDS. No cytogenetic abnormalities were detected. The patient was treated with intensive chemotherapy with initial response. However, blasts emerged 7 months later with the acquisition of a novel translocation t(5;21)(q13;q22), indicating disease transformation to AML (Additional file 1: Methods and Materials). The patient then underwent cord blood transplantation but the disease relapsed 9 months afterwards. Cytogenetic studies showed the same karyotype 46,XY,t(5;21)(q13;q22)[20] as in the MDS-transformed AML sample (Fig. 1a). Metaphase fluorescence in situ hybridization (FISH) analysis of the relapsed AML BM revealed splitting of *RUNX1* (Fig. 1b). However, no *RUNX1* fusion was detected in this sample by RNA-Seq (Illumina TruSight RNA Pan-Cancer Panel). Whole genome sequencing (WGS) on the relapsed AML revealed that chromosome 21 broke at intron 1 of *RUNX1*, whereas chromosome 5 broke at an intergenic region that is ~ 90 kb centromeric of *SERF1A* (Fig. 1c). The 5q13 breakpoint resides in the 500-kb inverted repeat at the spinal muscular atrophy locus, which is prone to rearrangements. Both the 21q22 and 5q13 breakpoints were confirmed to be identical in the MDS-transformed and relapsed AML samples (Fig. 1d). The 21q22 breakpoint is ~ 20 kb upstream of the P2 promoter, and therefore, the translocation is expected to remove P1, exon 1 and a large portion of intron 1, leaving P2 and downstream *RUNX1* exons intact on the derivative chromosome. The first intron of *RUNX1* has long been suggested to harbor *cis*-regulatory elements (CREs) and its disruption may perturb *RUNX1* expression [1–3]. Quantitative RT-PCR revealed markedly elevated expression of the P2 transcripts (*RUNX1b*/*RUNX1a*) in the relapsed AML BM, as compared to the t(5;21)-negative MDS and post-transplant samples from the patient (Fig. 1e). P1-derived *RUNX1c* was also upregulated. These increases were not due to *RUNX1* gain as WGS indicated a normal copy number in the relapsed sample. Also, analyses of 14 pairs of leukemia/remission BM samples from pediatric AML patients (7 with t(8;21)(q22;q22)/*RUNX1-RUNX1T1* with *RUNX1* breakage at intron 5 and 7 with other cytogenetic aberrations or a normal karyotype at diagnosis) revealed only modest *RUNX1* (overall *RUNX1a*/*RUNX1b*/*RUNX1c*) upregulation in the leukemia counterparts (mean 2.47-fold vs. 8.53-fold in our case) (Additional file 2: Figure S1). Together, these observations implicate that there exist negative regulatory sequences upstream of P2 and their disruption by the t(5;21) aberrantly upregulates *RUNX1*.

**Fig. 2:**
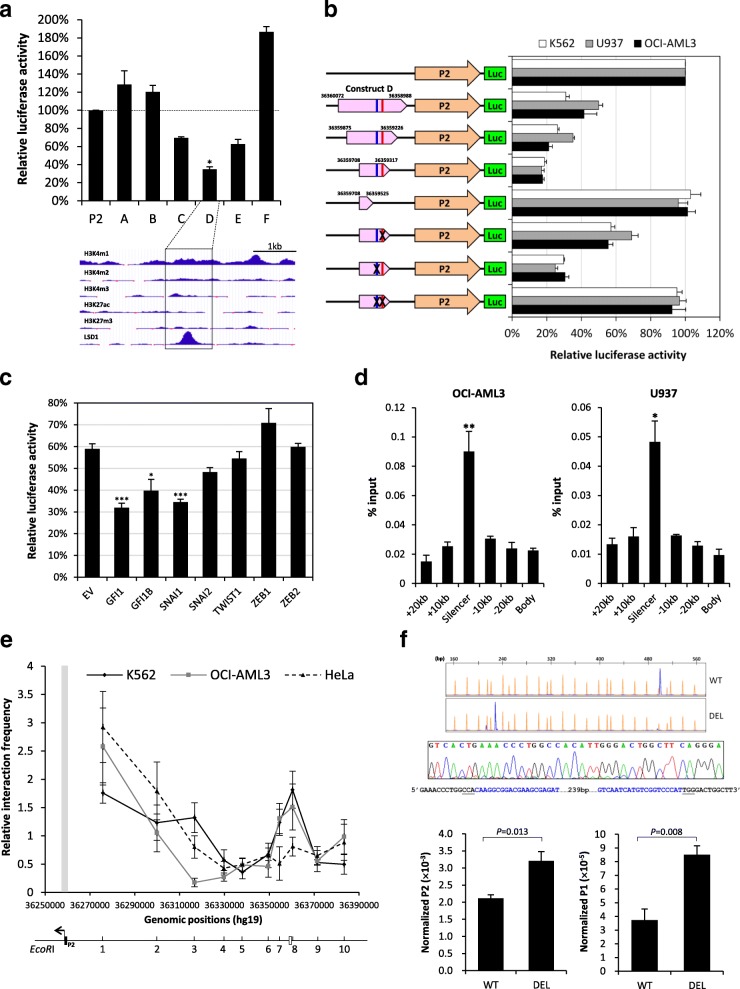
Functional characterization of the *RUNX1* intronic silencer. **a** Top, DHS fragments (~ 1 kb) were cloned upstream of P2 into pNL1.1. The resultant constructs were co-transfected with pGL4.54 into K562 cells. Results were compared to the pNL1.1-P2 control. Bottom, ENCODE ChIP-seq data at the repressive DHS in K562 cells, obtained from the UCSC genome browser. **b** Various deletion/mutant constructs were co-transfected with pGL4.54 into the cells. Blue and red lines represent the GFI1/GFI1B and SNAI1 motifs, respectively. Numbers indicate the genomic positions. Data are presented as in panel a. **c** The 392-bp silencer was cloned into pNL3.1 and the construct was co-transfected with pGL4.54 and pCI vectors expressing different transcription factors (TF) into HeLa cells. Parallel experiments using empty pNL3.1 were performed for each TF group. Results were compared to the empty pCI group (EV). **d** ChIP-qPCR analysis of LSD1 binding to the silencer. Results were compared to flanking intron 1 regions (20 kb from the silencer) as well as exon 5 (Body) of *RUNX1*. In panels a, c and d, data were analyzed by one-way ANOVA followed by Dunn’s test. *, ** and *** indicate *p* < 0.05, *P* < 0.01 and *P* < 0.001, respectively. **e** 3C analysis of chromatin interactions between P2 and the silencer. A physical map of the 10 analyzed *EcoR*I sites is also shown. The open box represents the silencer. In panels a-e, data are expressed as mean ± SE from three independent experiments. **f** CRISPR/Cas9 disruption of the silencer. Top, a representative cell population (DEL) showing biallelic deletion of the silencer. WT represents the wild-type genotype. The deletion was verified by Sanger sequencing. The two guide RNA (blue) flanking the target region is shown and the PAM sites are underlined. Bottom, *RUNX1* P2 (*RUNX1b*/*RUNX1a*) and P1 (*RUNX1c*) transcript expression in cell populations with (DEL) (*n* = 6) or without (WT) (*n* = 6) deletion of the silencer. Data were analyzed by the Mann-Whitney test

Analyses of the ENCODE ChIA-PET (Chromatin Interaction Analysis by Paired-End Tag Sequencing) RNA polymerase II data from K562 cells revealed four regions in intron 1 that showed robust interactions with the P2 promoter (Additional file 3: Table S2). All these regions contain DNaseI hypersensitive sites (DHS), suggesting potential regulatory functions. Luciferase reporter assays showed that the DHS at region 3 strongly repressed the P2 promoter (Fig. 2a). Further analyses of this region revealed a 392-bp silencer element (chr21:36359317–36,359,708) containing two conserved binding sites for the SNAG repressors GFI1/GFI1B and SNAI1 that contribute to the repression (Fig. 2b; Additional file 4: Figure S2). Overexpression of GFI1, GFI1B and SNAI1 but not the related SNAI2, TWIST1, ZEB1 and ZEB2 augmented the repression (Fig. 2c). Conversely, their knockdown alleviated the effect (Additional file 5: Figure S3). Both GFI1/GFI1B and SNAI1 recruit the histone demethylase LSD1/KDM1A to mediate transcriptional repression. Consistently, we found LSD1 occupancy at the silencer element in K562, OCI-AML3 and U937 cells (Fig. 2a, d). Treatment of the LSD1 inhibitor tranylcypromine upregulated *RUNX1* (Additional file 6: Figure S4), further implicating a role of LSD1 in the repression. Indeed, previous mouse studies have shown that Lsd1 is a crucial epigenetic regulator of hematopoiesis by repressing key hematopoietic stem/progenitor cell genes including *Runx1* [4]. Notably, the repressive effect of the silencer on P2 was found to reduce substantially when these elements were aligned oppositely (Additional file 7: Figure S5A). Also, the repression was found to be considerably weaker on P1 than P2 (Additional file 7: Figure S5B). Moreover, no direct interactions between the silencer and P1 were noted in the K562 ChIA-PET data. These observations suggest that the silencer is P2-specific and orientation-dependent, further substantiating the non-redundancy of the two *RUNX1* promoters. Chromosome conformation capture analysis revealed long-range chromatin interactions between P2 and the silencer in K562 and OCI-AML3 but not HeLa cells (Fig. 2e). Disruption of the silencer by CRISPR/Cas9 significantly upregulated P2- and P1-derived transcripts in OCI-AML3 (Fig. 2f). This line was selected because of its normal *RUNX1* copy number as revealed from public database and our FISH analysis (data not shown). Collectively, these findings indicate that the *RUNX1* P2 promoter is restrained by an intronic silencer involving SNAG repressors and their corepressor LSD1 in myeloid cells.

**Fig. 3:**
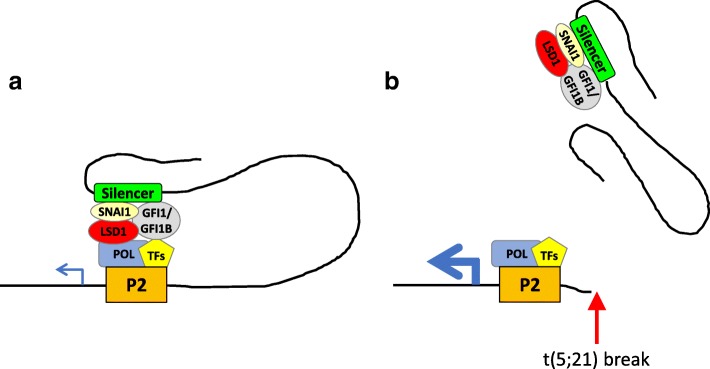
Molecular consequence of the novel t(5;21)(q13;q22) translocation. **a** The *RUNX1* P2 promoter is restrained by a long-range intronic silencer involving SNAG repressors and their corepressor LSD1 for tightly regulated gene expression in myeloid cells. **b** The t(5;21) breaks *RUNX1* upstream of the P2 promoter. This will disrupt the long-range transcriptional control, resulting in unrestrained P2 expression. It is possible that the increased P2 expression activates the P1 promoter on the native chromosome through a positive feedback loop, leading to the concomitant increase in P1 transcription. POL, RNA polymerase; TFs, transcription factors

The *RUNX1* breakpoints in nearly all the characterized *RUNX1* translocations in myeloid neoplasms fall downstream of the Runt homology domain [5]. t(5;21)(q13;q22) has been reported in 3 adults with myeloid neoplasms but the *RUNX1* breakpoint has only been mapped in one case to intron 6 [5]. In this study of a pediatric AML with t(5;21)(q13;q22), we found that *RUNX1* breaks at intron 1, which is upstream of the Runt domain separating the two *RUNX1* promoters. This region is involved in another *RUNX1* translocation t(12;21)(p13;q22)/*ETV6*-*RUNX1* found in B-lineage acute lymphoblastic leukemia. It was suggested that disruption of this intron might deregulate the *RUNX1* promoters and cause *RUNX1* overexpression in *ETV6-RUNX1*-positive patients without concurrent *RUNX1* gain [6]. Likewise, deregulated *RUNX1* transcription was suggested in a cryptic t(16;21)(p13;q22) involving breakage of the same intron in a familial platelet disorder (FPD)/AML case [3]. Here, we identified a silencer in this intron that interacts with the P2 promoter over a 100 kb distance. Since the *RUNX1* breakpoint in our case lies between the silencer and P2, the t(5;21) will disrupt this long-range control, resulting in unrestrained P2 transcription. It has been demonstrated that RUNX1 binds to two RUNX motifs in the P1 promoter to activate its transcription in hematopoietic cells [7]. It is thus possible that the increased P2-isoform expression might activate P1 on the native chromosome through a positive feedback loop, leading to the concomitant *RUNX1c* increase in our case (Fig. 3).

*WT1* (*Wilms tumor 1*) mutations are rare in MDS but associated with AML transformation [8]. We found *WT1* mutations in the two AML but not the MDS samples (Additional file 8: Figure S6). These findings suggest that *WT1* mutations and aberrant *RUNX1* upregulation by the t(5;21) might cooperatively contribute to the AML transformation. Concordantly, we found that the overall *RUNX1* expression is significantly increased in AML than MDS patients (Additional file 9: Figure S7). Also, a recent study has suggested that overexpression of the short *RUNX1* isoform by splicing factor mutations may be important in MDS progression [9]. In fact, it has been demonstrated that RUNX1 promotes the growth/survival of leukemic cells and is required for leukemogenesis [9, 10]. Prognostically, high *RUNX1* expression confers poor outcomes in leukemia patients [10].

It remains to be determined if additional CREs and/or factors are involved in the LSD1-mediated *RUNX1* repression. Also, different breakpoint locations within the first intron may impact *RUNX1* differentially. Due to scanty patient materials, the impact of the t(5;21) on neighboring 21q22 and 5q13 genes could not be assessed.

In conclusion, our findings uncover a novel mechanism of *RUNX1* deregulation by revealing that chromosomal translocations can also aberrantly upregulate *RUNX1*. This highlights the multifaceted impacts of chromosomal aberrations on *RUNX1* and may open a new avenue for investigating *RUNX1* alterations. Also, our findings support the emerging oncogenic role of RUNX1 in AML, suggesting that blocking RUNX1 activity may be a potential therapeutic approach. Lastly, the characterization of the *RUNX1* silencer further underscores the complex transcriptional control of the gene and provides new insights into CRE functions.

## Additional files

Additional file 1: **Methods and Materials.** including primers used in this study (Table S1) and supplementary references. (DOCX 32 kb)

Additional file 2: **Figure S1.** *RUNX1* expression in 14 pairs of leukemia/remission BM samples from pediatric AML patients. (DOCX 304 kb)

Additional file 3: **Table S2.** Regions of intron 1 that interact with the *RUNX1* P2 promoter as revealed from the K562 RNA polymerase II ChIA-PET data. (DOCX 26 kb)

Additional file 4: **Figure S2.** Multiple alignments (Multiz) of the putative GFI1/GFI1B and SNAI1 binding sites in the silencer element. (DOCX 307 kb)

Additional file 5: **Figure S3.** Knockdown of GFI1/GFI1B and SNAI1 alleviated the repressive effect of the silencer element. (DOCX 291 kb)

Additional file 6: **Figure S4.** Dose-dependent upregulation of *RUNX1* expression by the LSD1 inhibitor tranylcypromine (TCP). (DOCX 35 kb)

Additional file 7: **Figure S5.** Orientation- and promoter-dependent properties of the *RUNX1* intronic silencer. (DOCX 507 kb)

Additional file 8: **Figure S6.** *WT1* mutations in the AML but not MDS samples of the patient. (DOCX 1483 kb)

Additional file 9: **Figure S7.** *RUNX1* mRNA levels in MDS and AML patients. (DOCX 61 kb)

## Abbreviations

AML: Acute myeloid leukemia
BM: Bone marrow
ChIA-PET: Chromatin Interaction Analysis by Paired-End Tag Sequencing
CRE: *cis*-regulatory element
DHS: DNaseI hypersensitive sites
FISH: Fluorescence in situ hybridization
kb: kilobases
MDS: Myelodysplastic syndrome
WGS: Whole genome sequencing
