# Supplementary material for: RUNX1 upregulation via disruption of long-range transcriptional control by a novel t(5;21)(q13;q22) translocation in acute myeloid leukemia
Source: Mol Cancer. 2018 Aug 29;17:133. doi: 10.1186/s12943-018-0881-2 (PMC6116564; doi:10.1186/s12943-018-0881-2)
Supplement: Supplementary file 1 — Methods and Materials. including primers used in this study (Table S1) and supplementary references. (DOCX 32 kb) [file 12943_2018_881_MOESM1_ESM.docx]

**Methods and Materials**

***Patient samples and cell culture***

Bone marrow (BM) mononuclear cells were prepared from patient samples by density gradient centrifugation on Ficoll-Hypaque (GE Healthcare) and used for molecular studies.

OCI-AML3 was provided by Prof. M.D. Minden (Princess Margaret Cancer Centre, University Health Network, Toronto, Canada). Other cell lines were obtained commercially. Cells were maintained in RPMI-1640 medium containing 10% fetal bovine serum.

***Cytogenetic studies***

Cytogenetic studies were performed on Giemsa-banded metaphases obtained through short-term synchronized and unsynchronized cultures of BM cells supplemented by a direct harvest, according to standardized protocols previously published [1]. Briefly, air-dried slides were prepared following hypotonic treatment (0.075M KCl) and acetic acid/methanol (1:3) fixation. Prior to banding, the slides were heated at 60°C overnight and put in 7.5% H_2_O_2_ for 3 minutes. The metaphase chromosome preparation was G-banded by 0.05% trypsin and stained with Leishman’s stain for 3 minutes. At least 20 consecutive metaphases were analyzed. Details of the karyotype are reported in accordance with the International System for Human Cytogenomic Nomenclature (ISCN 2016) [2].

***FISH studies***

Metaphases were hybridized with the Vysis *ETV6*/*RUNX1* extra signal (ES) dual color translocation probe (Abbott Molecular), with the *ETV6* probe directly labeled with SpectrumGreen and the *RUNX1* probe with SpectrumOrange. The hybridization procedures were in accordance with protocols from the manufacturer. Briefly, pre-digestion of cells on each slide was carried out in 0.05% pepsin in 0.01M HCl. The slide and probes were then co-denatured at 72°C for 2 minutes, followed by overnight incubation at 37°C in a dark humidity chamber. The slide was washed and mounted in DAPI II counterstain (Abbott Molecular). Metaphase FISH on abnormal G-banded metaphases that were re-located using microscope co-ordinates, thus allowing direct correlation of FISH results with abnormalities on G-banding.

***Whole genome sequencing (WGS) and RNA-Seq***

For WGS, DNA library was prepared using the Illumina Nano DNA Library Prep Kit and sequenced on an Illumina HiSeq X sequencer. The Issac Aligner (version 01.15.02.08) was used to align the sequence reads to the human reference genome (hg19). Single nucleotide variants and small indels were called and annotated using Issac Variant Caller (version 2.0.13) and SnpEff (version 3.3). Structural and copy number variants were called by Manta (version 0.20.2) and Control-FREEC (version 6.4), respectively. The library preparation and all data analyses were performed by Macrogen Inc. A mean depth of 35× was achieved and 96% of target regions were covered by more than 20×. Primer sequences used for confirmation of the genomic breakpoints on der(21) and der(5) are provided in Table S1.

For RNA-Seq, the Illumina TruSight RNA Pan-Cancer Panel (targeting 1,385 cancer genes) was used to identify possible *RUNX1* fusions. Libraries were sized on QIAxcel Advanced using QIAxcel DNA screening kit (Qiagen) and quantified by real-time PCR. Sequencing was performed using MiSeq Reagent Kit v3 at 76 bp paired-end on an Illumina MiSeq sequencer. Data were analyzed by the RNA fusion analysis module (version 1.0.0.351) on Local Run Manager v2.0 (Illumina).

***Quantitative RT-PCR***

Total RNA was extracted by TRIZOL reagent (Thermo Fisher Scientific) and reverse-transcribed using the SuperScript III First-Strand Synthesis System (Thermo Fisher Scientific). Quantitative RT-PCR was performed using TaqMan Gene Expression Assays (*RUNX1b/c*: Hs00231079_m1; *RUNX1c*: Hs01021966_m1; *RUNX1a*: Hs04186042_m1; *SNAI1*: Hs00195591_m1; *GFI1*: Hs00382207_m1; *GFI1B*: Hs01062469_m1) (Thermo Fisher Scientific) on a 7300 Real-Time PCR System (Thermo Fisher Scientific). Each sample was measured in triplicate and *GAPDH* was used for normalization. Transcript levels were determined by the standard curve method. *RUNX1b* levels were calculated by subtracting total *RUNX1b*/*RUNX1c* from *RUNX1c*.

***Plasmid constructs, transient transfection and reporter gene assays***

Fragments of the human *RUNX1* P2 (969-bp, chr21:36260858-36261825) and P1 (1100-bp, chr21:36421584-36422683) promoters previously shown to be active in hematopoietic cells [3] were cloned into the *Bgl*II and *Hind*III sites of the NanoLuc luciferase reporter vector pNL1.1 (Promega). These fragments were found to yield similar luciferase activities in K562 (~80-fold) and U937 (~30-fold) cells as compared to the empty pNL1.1. Putative regulatory elements were then cloned upstream of the *RUNX1* promoters (*Sac*I/*Xho*I sites) into the resultant pNL1.1 constructs. Regulatory elements were also cloned into the NanoLuc luciferase vector pNL3.1, which carries a minimal TATA-box promoter (Promega). The GeneArt Site-Directed Mutagenesis System (Thermo Fisher Scientific) was used to mutate target sequences. Full-length cDNAs encoding SNAI1, SNAI2, TWIST1, ZEB1, ZEB2, GFI1 and GFI1B were cloned into the pCI expression vector (Promega). All cloning was performed using the In-Fusion HD Cloning Kit (Clontech). Primer sequences used for preparing reporter constructs are provided in Table S1.

K562 and HeLa cells were transfected by Lipofectamine 2000 (Thermo Fisher Scientific), whereas U937 and OCI-AML3 cells by the Viafect Transfection Reagent (Promega) in 96-well tissue culture plates according to the manufacturers’ instructions. Co-transfection with the Firefly luciferase reporter vector pGL4.54 was used for normalization of transfection efficiency. Each condition was tested in triplicate and the number of times the experiment was performed is indicated in the respective figure legends. Luciferase activities were measured using the Nano-Glo Dual-Luciferase Reporter Assay System (Promega) 48 hours after the transfection unless otherwise stated.

***Small interfering RNA (siRNA) knockdown***

K562 or U937 cells (5×10^6^) were electroporated with 1μM of Silencer Select Validated siRNA (Thermo Fisher Scientific) in 0.4 cm-gap cuvettes using the Gene Pulser Xcell Electroporation System (Bio-Rad) (300V, 950μF for K562 and 300V, 750μF for U937). Cells were allowed to recover for 24 hours before plasmid transfection.

***Chromatin immunoprecipitation (ChIP)***

ChIP assays were performed using the SimpleChIP Plus Sonication Chromatin IP Kit (Cell Signaling Technology) according to the manufacturer’s protocol. Briefly, cells (~1×10^7^) were fixed with 1% formaldehyde at room temperature for 20 minutes and chromatin was fragmented by the Branson SFX150 Sonifier. Sonicated chromatin was immunoprecipitated with 10μg of a ChIP-validated LSD1 antibody (Bethyl Laboratories, A300-215A) at 4°C overnight. Immunoprecipitated complexes were washed, eluted, and the cross-linking was reversed by heating the samples at 65°C for 2 hours. DNA was purified and analyzed by real-time PCR in triplicate using primers located at different regions of the *RUNX1* gene. Results from three independent experiments were averaged and presented. Primer sequences are provided in Table S1.

***Chromosome conformation capture (3C) analysis***

*EcoR*I-digested 3C libraries were prepared as previously described [4]. Briefly, formaldehyde-fixed nuclei prepared from 1×10^7^ cells were digested with *EcoR*I overnight, followed by ligation with T4 DNA ligase at 16°C for 3 hours and then room temperature for 30 minutes. The cross-linking was reversed and DNA was purified. A control library using an equal molar amount of the bacterial artificial chromosomes (BACs) RP11-77G18 and RP11-177L11 encompassing the human *RUNX1* locus was prepared to normalize for varying primer efficiencies. 3C ligation products were analyzed in triplicate by SYBR Green quantitative PCR using a constant primer located at the P2 promoter on a LightCycler 480 system (Roche Life Science). Ten *EcoR*I sites along intron 1 of *RUNX1* were tested. The specificity of the amplifications was confirmed by melting curve analysis. The 3C signals were quantified using standard curves obtained from serial dilutions of the BAC control library and normalized to the *ERCC3* control. Results from three independent experiments were averaged and presented. Primer sequences are provided in Table S1.

***CRISPR/Cas9-deletion of the silencer***

The Alt-R CRISPR-Cas9 System (Integrated DNA Technologies, Inc.) was used to delete a 273-bp region within the silencer containing the GFI1/GFI1B and SNAI1 binding sites in OCI-AML3 cells. Two guide RNA flanking the target region were designed using the online tool <http://crispr.mit.edu/>. Two ribonucleoprotein complexes were prepared according to the manufacturer’s instructions and transfected into OCI-AML3 cells by the Gene Pulser Xcell Electroporation System (150V, 700μF using 0.2 cm-gap cuvette) at a final concentration of 1.5μM. Transfected cells were serially diluted and heterogeneous populations carrying >95% of biallelic deletions identified by fragment analysis were used for expression analyses. The identity of the deletions in these cell populations (carry predominantly the mutant DNA sequences) was confirmed by Sanger sequencing. Primers used for screening of deleted clones by fragment analysis were 5’ACTTCATTCTTCACCGCTGTCT3’ (FAM-labelled) and 5’CCCCTAACCTCAACCACTGA3’.

***Statistical analysis***

Statistical analysis was performed using GraphPad Prism 6 (GraphPad Software). Two-sided *P* values less than .05 were considered statistically significant.

**Supplementary references:**

1. Hui EK, Wan TS, Ng MH. Chromosome Preparation for Myeloid Malignancies. In: Wan TS. Cancer Cytogenetics: Methods and protocols. Methods Mol Biol. New York: Humana; 2017. p. 11-7.
2. McGowan-Jordan J, Simons A, Schmid M. An International System for Human Cytogenomic Nomenclature. Basel: S. Karger; 2016.
3. Ghozi MC, Bernstein Y, Negreanu V, Levanon D, Groner Y. Expression of the human acute myeloid leukemia gene AML1 is regulated by two promoter regions. Proc Natl Acad Sci U S A. 1996;93:1935-40.
4. Naumova N, Smith EM, Zhan Y, Dekker J. [Analysis of long-range chromatin interactions using Chromosome Conformation Capture.](https://www.ncbi.nlm.nih.gov/pubmed/22903059) Methods. 2012;58:192-203.
5. Nottingham WT, Jarratt A, Burgess M, Speck CL, Cheng JF, Prabhakar S, et al. Runx1-mediated hematopoietic stem-cell emergence is controlled by a Gata/Ets/SCL-regulated enhancer. Blood. 2007;110:4188-97.
6. Haferlach T, Kohlmann A, Wieczorek L, Basso G, Kronnie GT, Béné MC, et al. [Clinical utility of microarray-based gene expression profiling in the diagnosis and subclassification of leukemia: report from the International Microarray Innovations in Leukemia Study Group.](https://www.ncbi.nlm.nih.gov/pubmed/20406941) J Clin Oncol. 2010;28:2529-37.
7. Bagger FO, Sasivarevic D, Sohi SH, Laursen LG, Pundhir S, Sønderby CK, et al. [BloodSpot: a database of gene expression profiles and transcriptional programs for healthy and malignant haematopoiesis.](https://www.ncbi.nlm.nih.gov/pubmed/26507857) Nucleic Acids Res. 2016;44:D917-24.

**Table S1. Primers used in this study.**

| **Primer** | **Sequence (5’ to 3’)** | **Purpose** |
| --- | --- | --- |
| 21q22-F | ATTTGCAAACAGGCCCACTC | Genomic breakpoint confirmation |
| 5q13-R | TCCAATATGTTAGTTTTCTTTGATTTCT | Genomic breakpoint confirmation |
| 5q13-F | CCATTGACTGTAGGTAGGTCC | Genomic breakpoint confirmation |
| 21q22-R | CCCGAGGACATTTTGCACAT | Genomic breakpoint confirmation |
| pNL1.1-P2-F | GAGGATATCAAGATCTCGTGGCTGCTTTCAACTTT | Reporter construct |
| pNL1.1-P2-R | CCGGATTGCCAAGCTTGGTTGACTTCCTTCTGGC | Reporter construct |
| pNL1.1-P1-F | GAGGATATCAAGATCTGGGCCGGAAAATGAAATACG | Reporter construct |
| pNL1.1-P1-R | CCGGATTGCCAAGCTTTGAGGCCCAAAGAAGTTTTCA | Reporter construct |
| Construct A-F | ACTGGCCGGTACCTGAGCTCCACCAGTGAAATAGCTGTGCA | Reporter construct |
| Construct A-R | TCTTGATATCCTCGAGTTTAGGAAGTGGCTGCTGGG | Reporter construct |
| Construct B-F | ACTGGCCGGTACCTGAGCTCCCCAGGTAGCCAAGAATCAA | Reporter construct |
| Construct B-R | TCTTGATATCCTCGAGGTAGAGACGGGGTTTCACCA | Reporter construct |
| Construct C-F | ACTGGCCGGTACCTGAGCTCTCTGCCCTCATATGGGACCT | Reporter construct |
| Construct C-R | TCTTGATATCCTCGAGATCTCTTTTGTGTGCTGGGAGT | Reporter construct |
| Construct D-F | ACTGGCCGGTACCTGAGCTCGGTTCTGCCGAATTCCTGTT | Reporter construct |
| Construct D-R | TCTTGATATCCTCGAGCAAGGGATGTCAAGGGTTGC | Reporter construct |
| Construct E-F | ACTGGCCGGTACCTGAGCTCGACCCATAGGAGGCCAAAAT | Reporter construct |
| Construct E-R | TCTTGATATCCTCGAGAGATGAGAGGTGTGCGGAGT | Reporter construct |
| Construct F-F | ACTGGCCGGTACCTGAGCTCGGTGGCTCCAGTTAAACCAG | Reporter construct |
| Construct F-R | TCTTGATATCCTCGAGGGGGACTTGTTGGTGGAT | Reporter construct |
| ChIP-body-F | CCACCAACCTCATTCTGTTT | ChIP |
| ChIP-body-R | AGACATGGTCCCTGAGTATA | ChIP |
| ChIP-down2-F | AAGCTCAATGGGAAAACGCT | ChIP |
| ChIP-down2-R | ATCGCTTGAAACTGGGAGGT | ChIP |
| ChIP-down1-F | TTGATGCTCACCCTCACACT | ChIP |
| ChIP-down1-R | AGGCTACCAGTGTTTCCTGT | ChIP |
| ChIP-silencer-F | CGAGCCTAGTCAAAACAACGT | ChIP |
| ChIP-silencer-R | AAGCCTCACCCCACAAGG | ChIP |
| ChIP-up1-F | AAGACGACTTCCAGTGCCAA | ChIP |
| ChIP-up1-R | TGGCCTTGAGTCCACAGAAT | ChIP |
| ChIP-up2-F | CACACGACCTGCTCTCCTAA | ChIP |
| ChIP-up2-R | TGATCCAAAGGGAAGAAAGG | ChIP |
| 3C-anchor | CTGGGCCTTCTTATGATTGTTGT | 3C |
| 3C-1 | GTGATTCGGCACCCAAAAT | 3C |
| 3C-2 | GCAAATTCGAGGCAAGGGAAC | 3C |
| 3C-3 | TGGCTGAAGAAGTATCACATGG | 3C |
| 3C-4 | CTGGAGAGGTACACAGGCAC | 3C |
| 3C-5 | GTGTGTGTGCTCTGTGTCAC | 3C |
| 3C-6 | CATCGATCTTTGGGTACAGTGG | 3C |
| 3C-7 | GGTAGTTCCCTGGGTAGCTTG | 3C |
| 3C-8 | GAGGGGCGCATACTGACT | 3C |
| 3C-9 | TCTGCCCAGTTGTGTGTGC | 3C |
| 3C-10 | AGCATTTTGATTTTGGTAGTGGT | 3C |
| ERCC3-F1 | CCGTGACAACTGAGATGGCT | 3C |
| ERCC3-F2 | GGAGGAACAACTGCCCCATA | 3C |
