# Supplementary material for: RUNX1 upregulation via disruption of long-range transcriptional control by a novel t(5;21)(q13;q22) translocation in acute myeloid leukemia
Source: Mol Cancer. 2018 Aug 29;17:133. doi: 10.1186/s12943-018-0881-2 (PMC6116564; doi:10.1186/s12943-018-0881-2)
Supplement: Supplementary file 2 — Figure S1. RUNX1 expression in 14 pairs of leukemia/remission BM samples from pediatric AML patients. (DOCX 304 kb) [file 12943_2018_881_MOESM2_ESM.docx]

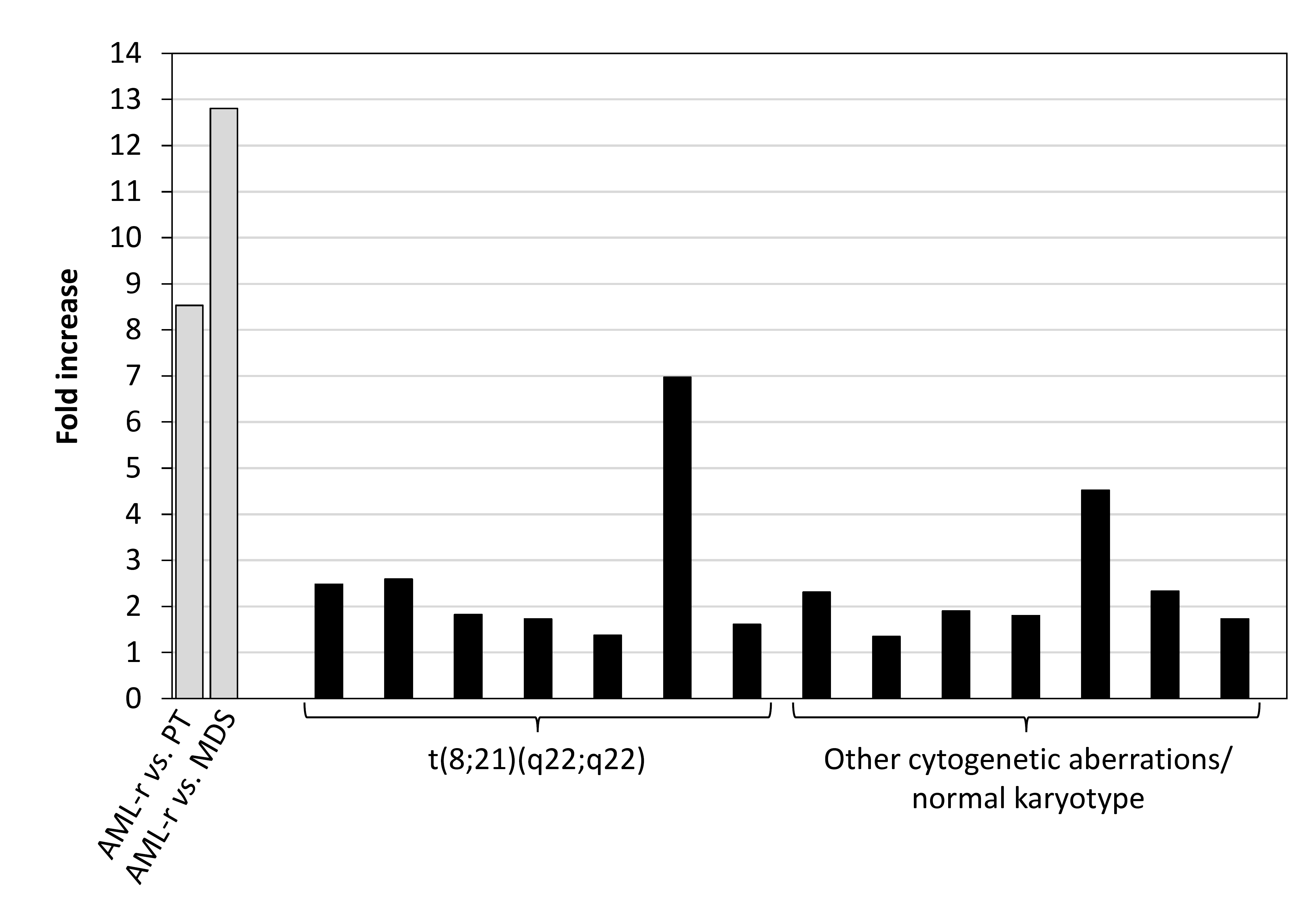


**Figure S1. *RUNX1* expression in 14 pairs of leukemia/remission BM samples from pediatric AML patients.** The cohort includes 7 patients with t(8;21)(q22;q22)/*RUNX1-RUNX1T1* and 7 with other cytogenetic aberrations or a normal karyotype at diagnosis. *RUNX1* mRNA levels (overall *RUNX1a*/*RUNX1b*/*RUNX1c*) were determined by quantitative RT-PCR and normalized to *GAPDH*. Results are expressed as fold increase by comparing the mRNA level at diagnosis to that at remission. The two grey bars show the fold increases in our t(5;21) case. AML-r, relapsed AML BM; PT, post-transplant BM; MDS, initial MDS BM.
