# Supplementary material for: RUNX1 upregulation via disruption of long-range transcriptional control by a novel t(5;21)(q13;q22) translocation in acute myeloid leukemia
Source: Mol Cancer. 2018 Aug 29;17:133. doi: 10.1186/s12943-018-0881-2 (PMC6116564; doi:10.1186/s12943-018-0881-2)
Supplement: Supplementary file 3 — Table S2. Regions of intron 1 that interact with the RUNX1 P2 promoter as revealed from the K562 RNA polymerase II ChIA-PET data. (DOCX 26 kb) [file 12943_2018_881_MOESM3_ESM.docx]

**Table S2. Regions of intron 1 that interact with the *RUNX1* P2 promoter as revealed from the K562 RNA polymerase II ChIA-PET data.**

| Region* | Genomic locations (hg19) | Size (bp) | DHS in K562 | Reporter construct |
| --- | --- | --- | --- | --- |
| 1 | 36317263-36322007 | 4744 | 36319161-36319310 | A |
| 2 | 36354615-36357007 | 2392 | 36355481-36355630  36356141-36356290 | B |
|  |  |  |  | C |
| 3 | 36357230-36360715 | 3485 | 36359421-36359570 | D |
| 4 | 36395588-36399925 | 4337 | 36398421-36398570  36399141-36399290^#^  36399521-36399670^#^ | E |
|  |  |  |  | F |

DHS, DNaseI hypersensitive sites obtained from the ENCODE project.

* Regions that were commonly detected and contained > 5 PETs in the two replicates in the ChIA-PET data.

^#^ The two DHS are closely spaced and cloned into the same reporter construct (Construct F). This fragment contains the human equivalent of the mouse +23 *Runx1* intronic enhancer [5].
