# Supplementary material for: RUNX1 upregulation via disruption of long-range transcriptional control by a novel t(5;21)(q13;q22) translocation in acute myeloid leukemia
Source: Mol Cancer. 2018 Aug 29;17:133. doi: 10.1186/s12943-018-0881-2 (PMC6116564; doi:10.1186/s12943-018-0881-2)
Supplement: Supplementary file 4 — Figure S2. Multiple alignments (Multiz) of the putative GFI1/GFI1B and SNAI1 binding sites in the silencer element. (DOCX 307 kb) [file 12943_2018_881_MOESM4_ESM.docx]

**Figure S2. Multiple alignments (Multiz) of the putative GFI1/GFI1B and SNAI1 binding sites in the silencer element.** The consensus GFI1/GFI1B (*left*) and SNAI1 (*right*) binding sites are shown. The red boxes indicate the core motifs. Both motifs are highly conserved across the mammalian species except opossum. The GFI1/GFI1B motif is also conserved in frog (*X. tropicalis*).
