# Supplementary material for: RUNX1 upregulation via disruption of long-range transcriptional control by a novel t(5;21)(q13;q22) translocation in acute myeloid leukemia
Source: Mol Cancer. 2018 Aug 29;17:133. doi: 10.1186/s12943-018-0881-2 (PMC6116564; doi:10.1186/s12943-018-0881-2)
Supplement: Supplementary file 5 — Figure S3. Knockdown of GFI1/GFI1B and SNAI1 alleviated the repressive effect of the silencer element. (DOCX 291 kb) [file 12943_2018_881_MOESM5_ESM.docx]

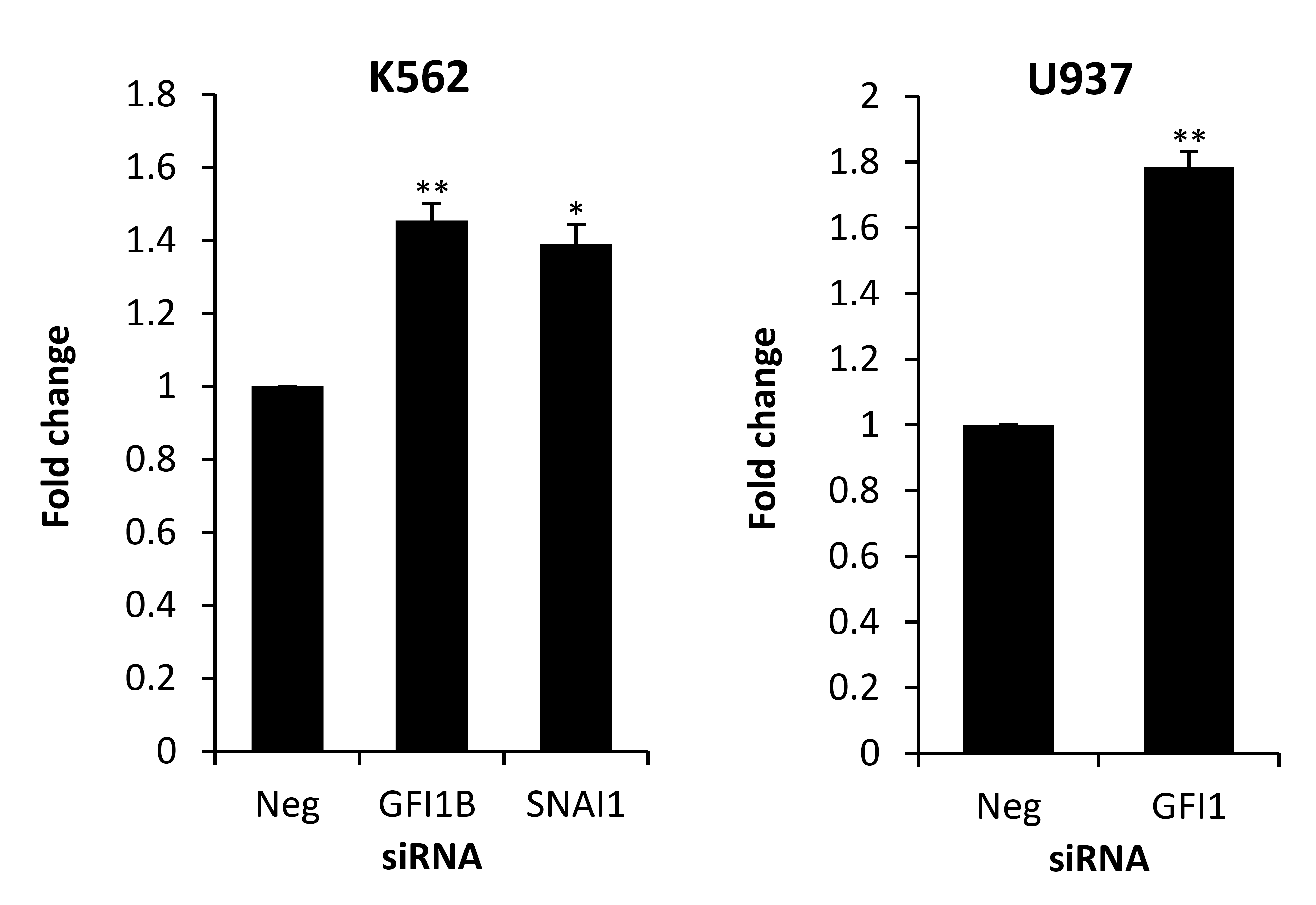


**Figure S3. Knockdown of GFI1/GFI1B and SNAI1 alleviated the repressive effect of the silencer element.** The 392-bp silencer was cloned upstream of the P2 promoter into pNL1.1. The resultant construct was co-transfected with pGL4.54 into K562 cells pre-treated with the indicated siRNA. Parallel transfection experiments using the pNL1.1-P2 promoter control were performed to calculate the levels of repression. Luciferase activities were measured 24 hours post transfection. Results are presented as fold change by comparing the repression to the negative siRNA-treated group (Neg). The effect of *GFI1* knockdown was examined in U937 cells, which highly express the SNAG repressor. The knockdown efficiency for *GFI1*, *GFI1B* and *SNAI1* was 52.6±4.6%, 45.7±3.5% and 48.1±2.3%, respectively as determined by quantitative RT-PCR performed 48 hours after siRNA treatment. Data are expressed as mean±SE from 2 independent experiments each performed in triplicate. * and ** indicate *P*<0.05 and *P*<0.01 *vs.* Neg siRNA, respectively by the Mann-Whitney test.
