# Supplementary material for: RUNX1 upregulation via disruption of long-range transcriptional control by a novel t(5;21)(q13;q22) translocation in acute myeloid leukemia
Source: Mol Cancer. 2018 Aug 29;17:133. doi: 10.1186/s12943-018-0881-2 (PMC6116564; doi:10.1186/s12943-018-0881-2)
Supplement: Supplementary file 6 — Figure S4. Dose-dependent upregulation of RUNX1 expression by the LSD1 inhibitor tranylcypromine (TCP). (DOCX 35 kb) [file 12943_2018_881_MOESM6_ESM.docx]

*******

******

*******

******

******

******

**Figure S4. Dose-dependent upregulation of *RUNX1* expression by the LSD1 inhibitor tranylcypromine (TCP).** K562 (*Left*) and OCI-AML3 (*Right*) cells were treated with the indicated concentrations of TCP for 24 hours. *RUNX1b* and *RUNX1c* levels were determined by quantitative RT-PCR and normalized to *GAPDH*. Results are expressed as mean±SE from two independent experiments each performed in triplicate. ** and *** indicate *P*<0.01 and *P*<0.001 *vs.* the vehicle control, respectively. Data were analyzed by one-way ANOVA followed by Dunn’s test.
