# Supplementary material for: RUNX1 upregulation via disruption of long-range transcriptional control by a novel t(5;21)(q13;q22) translocation in acute myeloid leukemia
Source: Mol Cancer. 2018 Aug 29;17:133. doi: 10.1186/s12943-018-0881-2 (PMC6116564; doi:10.1186/s12943-018-0881-2)
Supplement: Supplementary file 7 — Figure S5. Orientation- and promoter-dependent properties of the RUNX1 intronic silencer. (DOCX 507 kb) [file 12943_2018_881_MOESM7_ESM.docx]

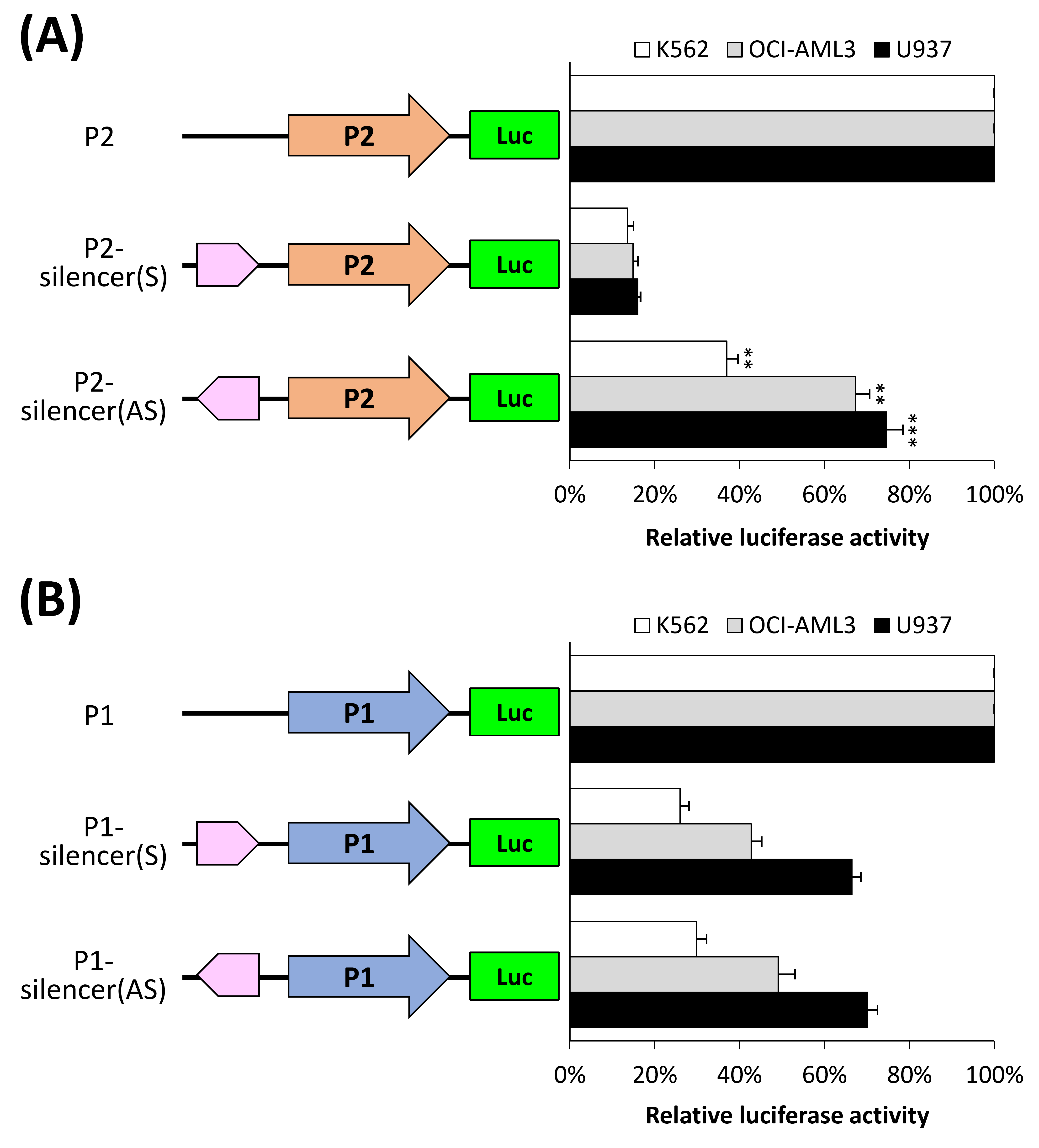


**Figure S5. Orientation- and promoter-dependent properties of the *RUNX1* intronic silencer.** (A) The 392-bp silencer element (*pink arrow*) was cloned in a sense (S) or anti-sense (AS) orientation upstream of the *RUNX1* P2 promoter into pNL1.1. The resultant constructs were co-transfected with pGL4.54 into different cell lines. Results are compared to the promoter control and expressed as mean±SE from three independent experiments. ** and *** indicate *P*<0.01 and *P*<0.001 *vs.* P2-silencer(S), respectively. (B) Parallel experiments were performed for the P1 promoter. The repressive effect of the silencer (cloned in the sense orientation) was significantly lower on the P1 than P2 promoter in the three cell lines tested (*P*<0.01 for K562 and OCI-AML3 and *P*<0.001 for U937). Also, no apparent orientation-dependent effect of the silencer on the P1 promoter was observed. Data were analyzed by the Mann-Whitney test.
