# Supplementary material for: RUNX1 upregulation via disruption of long-range transcriptional control by a novel t(5;21)(q13;q22) translocation in acute myeloid leukemia
Source: Mol Cancer. 2018 Aug 29;17:133. doi: 10.1186/s12943-018-0881-2 (PMC6116564; doi:10.1186/s12943-018-0881-2)
Supplement: Supplementary file 8 — Figure S6. WT1 mutations in the AML but not MDS samples of the patient. (DOCX 1483 kb) [file 12943_2018_881_MOESM8_ESM.docx]

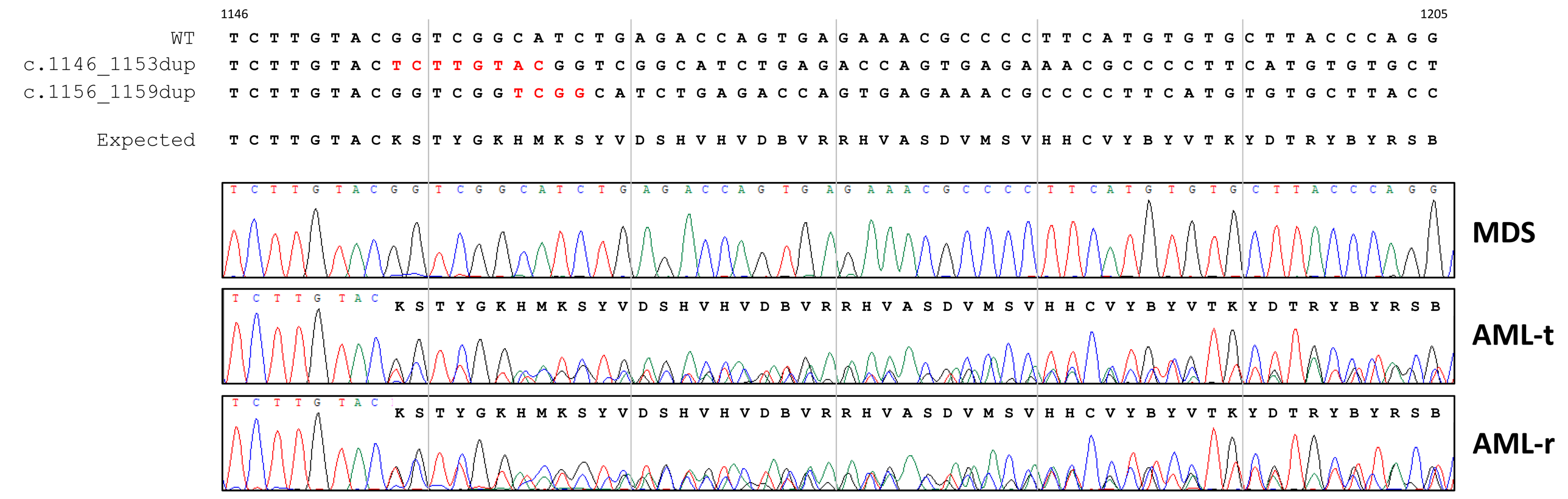


**Figure S6. *WT1* mutations in the AML but not MDS samples of the patient.** Two frameshift mutations (c.1146_1153dup/p.Arg385Leu*fs**72 and c.1156_1159dup/p.Ala387Val*fs**4) (variant allele frequency of 26% and 44%, respectively) were detected in exon 7 of the *WT1* gene in the relapsed AML BM sample (AML-r) by WGS. The nucleotide sequences of the wild-type (WT) and mutants are shown. The duplicated sequences are highlighted in *red*. The numbering refers to the coding sequence of NM_024426.5. Sanger sequencing indicated the expected mutations in AML-r. The same mutations were also detected in the MDS-transformed AML (AML-t) but not the initial MDS sample.
