# Supplementary material for: RUNX1 upregulation via disruption of long-range transcriptional control by a novel t(5;21)(q13;q22) translocation in acute myeloid leukemia
Source: Mol Cancer. 2018 Aug 29;17:133. doi: 10.1186/s12943-018-0881-2 (PMC6116564; doi:10.1186/s12943-018-0881-2)
Supplement: Supplementary file 9 — Figure S7. RUNX1 mRNA levels in MDS and AML patients. (DOCX 61 kb) [file 12943_2018_881_MOESM9_ESM.docx]

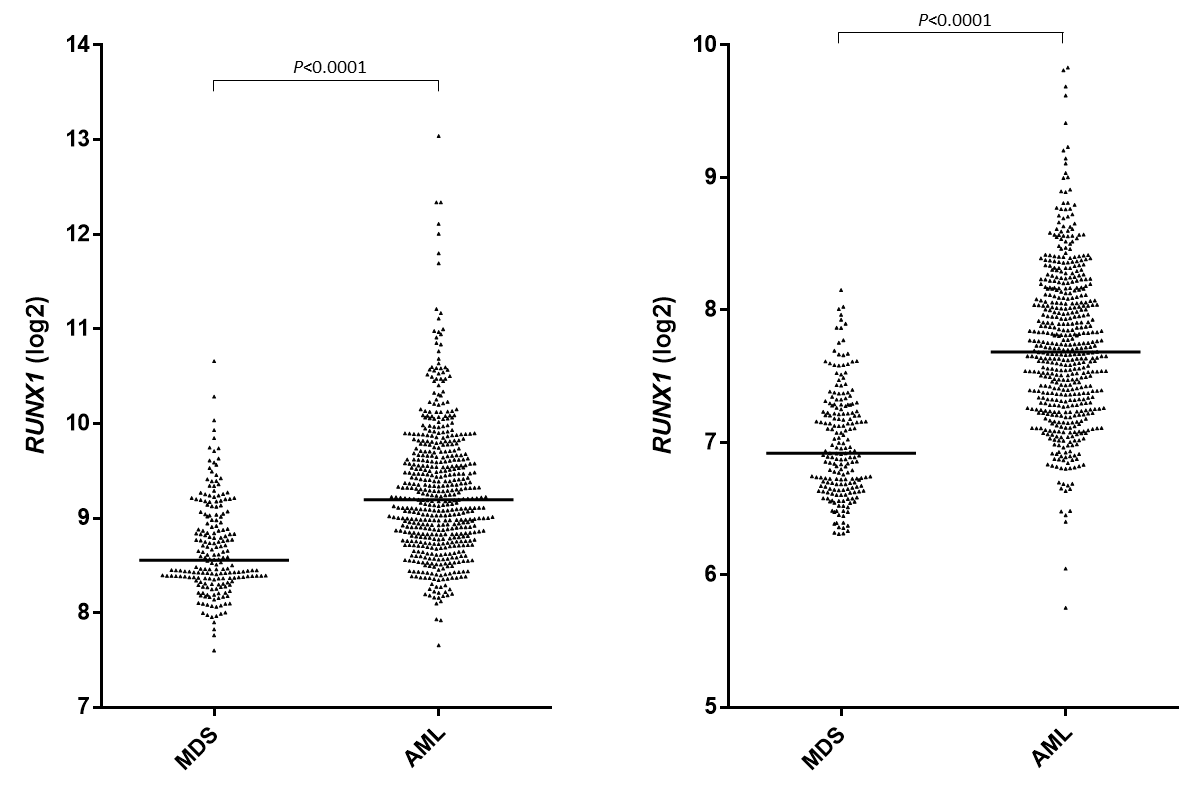


**Figure S7. *RUNX1* mRNA levels in MDS and AML patients.** *RUNX1* expression data from 206 MDS and 542 AML patients were obtained from the Leukemia MILE study (GSE13159) using BloodSpot [6,7]. Each triangle represents one patient and the horizontal line indicates the median level. *Left*, *RUNX1* expression from the max probe 211182_x_at (specific for *RUNX1a*/*RUNX1b*/*RUNX1c*). *Right*, mean overall expression of *RUNX1* obtained from all the probes in the dataset. Data were analyzed by unpaired t test.
